# Supplementary material for: Informational and Normative Influence on Conformity in Autism
Source: Autism Res. 2026 May 25;19(7):e70284. doi: 10.1002/aur.70284 (PMC13377261; doi:10.1002/aur.70284)
Supplement: Supplementary file 1 — Note S1. Preregistered group comparison of across‐trial conformity tendencies. Note S2. Group comparison of across‐trial conformity tendencies using GLMM analysis. Figure S1: Results of preregistered group comparison of measured variables. Figure S2: Preregistered group comparison of conformity tendencies. Figure S3: Exploratory analyses on the initial responses of the dot‐counting task. Table S1: Results of GLMMS1 examining group differences in across‐trial conformity tendencies. Table S2: Results of GLMM1 of random slope model examining group differences in conformity tendencies between the preference rating and dot‐counting tasks. [file AUR-19-0-s001.docx]

**Supporting Information**

**Supplementary Note 1: Preregistered group comparison of across-trial conformity tendencies**

To measure participants’ conformity tendency across-trial in the dot-counting task, we also conducted a multiple regression analysis for every participant individually, as follows:

Gap1D (*t* + 1) = 1 + β_3_ × Gap1O(t)… (S1)

In the equation (S1), *t* represents the number of trials, and *Gap1O*(t) denotes the difference between the participant’s first rating and the ratings from other individuals (other people’s answer – participant’s first answer) at trial *t*. *Gap1D*(*t* + 1) represents the difference between the participant’s first rating and actual number of dots (actual number of dots – first rating) at trial *t* + 1.　Then, we compared across-trial conformity tendencies (i.e., β_3_) between groups using a two-sample t-test. We also examined whether the across-trial conformity tendencies were different from 0 in each group using a one-sample t-test. We applied the conventional significance threshold *p* < .05.

**Supplementary Note 2: Group comparison of across-trial conformity tendencies using GLMM analysis**

To conduct a group comparison on across-trial conformity tendencies in the dot-counting task, we also constructed a GLMM, as follows:

Gap1D (*t* + 1) ~ 1 + Gap1O(*t*) + Gap1O(*t*):Group + (1 | Participant)…(GLMMS1)

where *t* represents the number of trials, and *Gap1O(t)* denotes the difference between the participant’s first rating and the ratings from other individuals (other people’s answer – participant’s first answer) at trial *t*. *Gap1D(t* *+* *1)* represents the difference between the participant’s first rating and actual number of dots (actual number of dots – first rating) at trial *t* + 1.


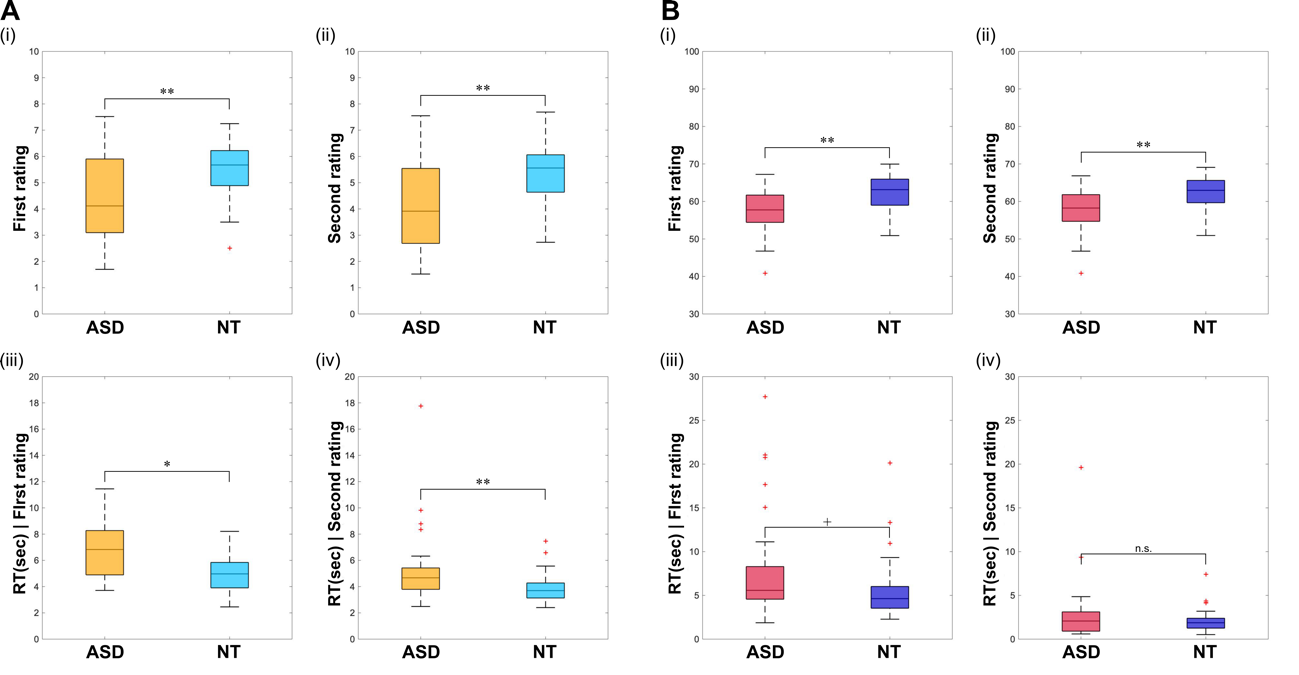


Figure S1 Results of preregistered group comparison of measured variables

A. Results of group comparison of measured variables in the preference rating task. (i) Participants’ first ratings of the preference rating task are illustrated. Individuals with ASD displayed significantly lower preferences than NT individuals for the second ratings (*t* (58) = −3.72, *p* = .001). (ii) Participants’ second ratings of the preference rating task are illustrated. Individuals with ASD reported significantly lower preferences than NT individuals for the second ratings (*t* (58) = −3.85, *p* = .001). (iii) Response times for the first ratings of the preference rating task are illustrated. Individuals with ASD responded significantly slower than NT individuals for the first rating (*t* (58) = 2.62, *p* = .014). (iv) Response times for the second ratings of the preference rating task are illustrated. Individuals with ASD responded significantly slower than NT individuals for the first rating (*t* (58) = 3.21, *p* = .003). B. Results of group comparison of measured variables in the dot-counting task. (i) Participants’ first ratings of the dot-counting task are illustrated. Individuals with ASD estimated the number of dots to be lower than NT individuals for the first ratings (*t* (58) = −2.97, *p* = .006). (ii) Participants’ second ratings of the dot-counting task are illustrated. Individuals with ASD estimated the number of dots to be significantly lower than NT individuals for the second ratings (*t* (58) = −2.86, *p* = .008). (iii) Response times for the first ratings of the dot-counting task are illustrated. Individuals with ASD took a marginally significantly longer time than NT individuals (*t* (58) = 1.94, *p* = .062). (iv) Response times for the second ratings of the dot-counting task are illustrated. Individuals with ASD determined their final answer (whether to maintain or revise their first response) as quickly as NT individuals (*t* (58) = 1.26, *p* = .219). The box indicates the interquartile range (25th–75th percentile), and the central line represents the median. Whiskers extend to 1.5 × IQR, and red crosses indicate outliers. ^∗∗^*p* < .001, ^∗^*p* < .05, + *p* < 0.1, n.s.: not significant.


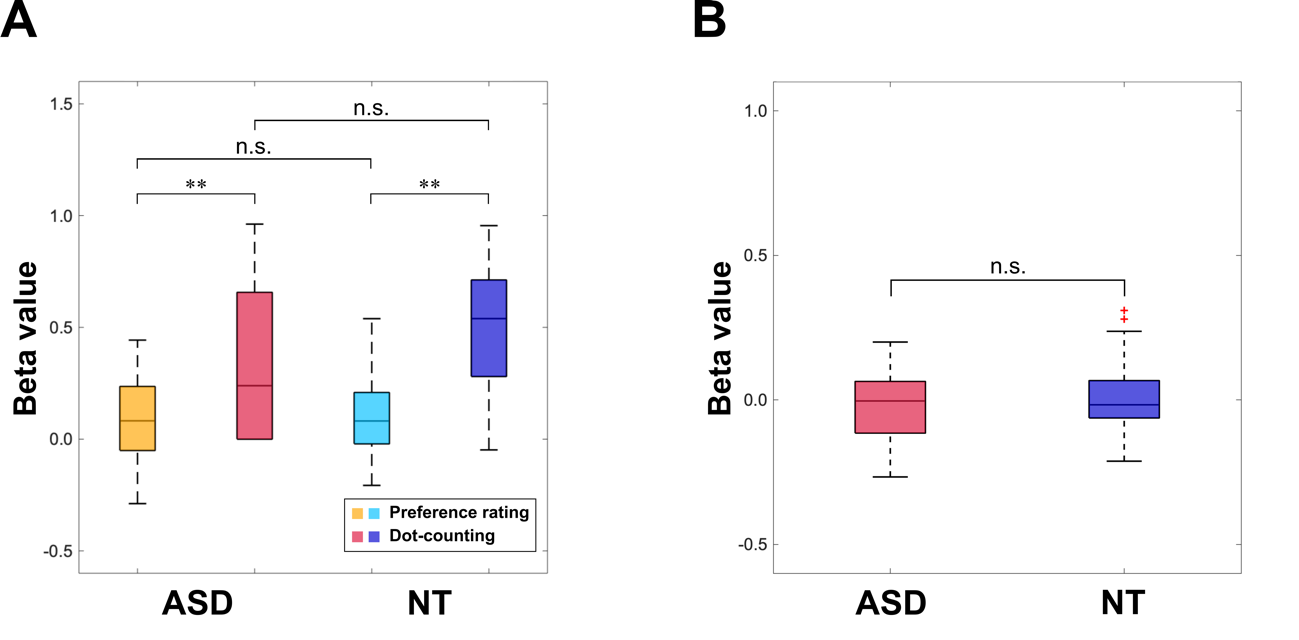


Figure S2 Preregistered group comparison of conformity tendencies

Results of the preregistered group comparison of conformity tendencies. (A) Results of the mixed two-way ANOVA on conformity tendencies. The conformity tendencies (calculated as β2 from Equation 1) were significantly greater than 0 in both groups across both tasks (preference rating: ASD: *t* (29) = 2.29, *p* = .029; NT: *t* (29) = 2.85, *p* = .008; dot-counting: ASD: *t* (29) = 5.54, *p* < .001; NT: *t* (29) = 9.79, *p* < .001). Subsequently, we compared participants’ conformity tendencies (β2) using a mixed two-way ANOVA, with group (ASD and NT) as a between-subject factor and task type (preference rating and dot-counting task) as a within-subject factor. As a result, we found marginally significant main effects of Group (*F* (1,58) = 3.23, *p* = .077, η_p_^2^ = .053) and Task (*F* (1,58) = 60.32, *p* < .001, η_p_^2^ = .51). The Group by Task interaction trended toward significance (*F* (1,58) = 2.89, *p* = .094, η_p_^2^ = .047). The simple main effects analysis further demonstrated that Task had a significant effect in both groups, indicating that conformity tendency in the dot-counting task was stronger than in the preference rating task in both groups (ASD: *F* (1,58) = 18.40, *p_adjusted_* < .001, η_p_^2^ = .241; NT: *F* (1,58) = 44.81, *p_adjusted_* < .001, η_p_^2^ = .436). However, the simple main effect of Group was not significant in either the preference rating or dot-counting task (preference rating：*F* (1,58) = 0.15, *p_adjusted_* = 1.000, η_p_^2^ =.002; dot-counting：*F* (1,58) = 4.22, *p_adjusted_* = .178, η_p_^2^ = .068). (B) Results of the group comparison of across-trial conformity tendencies in the dot-counting task. The across-trial conformity tendencies (calculated as β3 from Equation (S1)) in the dot-counting task were not different from 0 in both groups (ASD: *t* (29) = −0.69, p = .498; NT: *t* (29) = 0.49, *p* = .625). We did not find a significant group difference (*t* (58) = −0.75, *p* = .460). The box indicates the interquartile range (25th–75th percentile), and the central line represents the median. Whiskers extend to 1.5 × IQR, and red crosses indicate outliers. ∗∗*p* < .001, n.s.: not significant.

*Note.* In the preregistered analysis, the estimated group difference in the dot-counting task was ASD−NT = −0.17, 95% CI [−0.32, 0.00] (Hedges’ g = −0.52). The direction of this effect is consistent with that of the trial-level GLMM; the wider CI suggests reduced estimation precision in the summary-based approach.


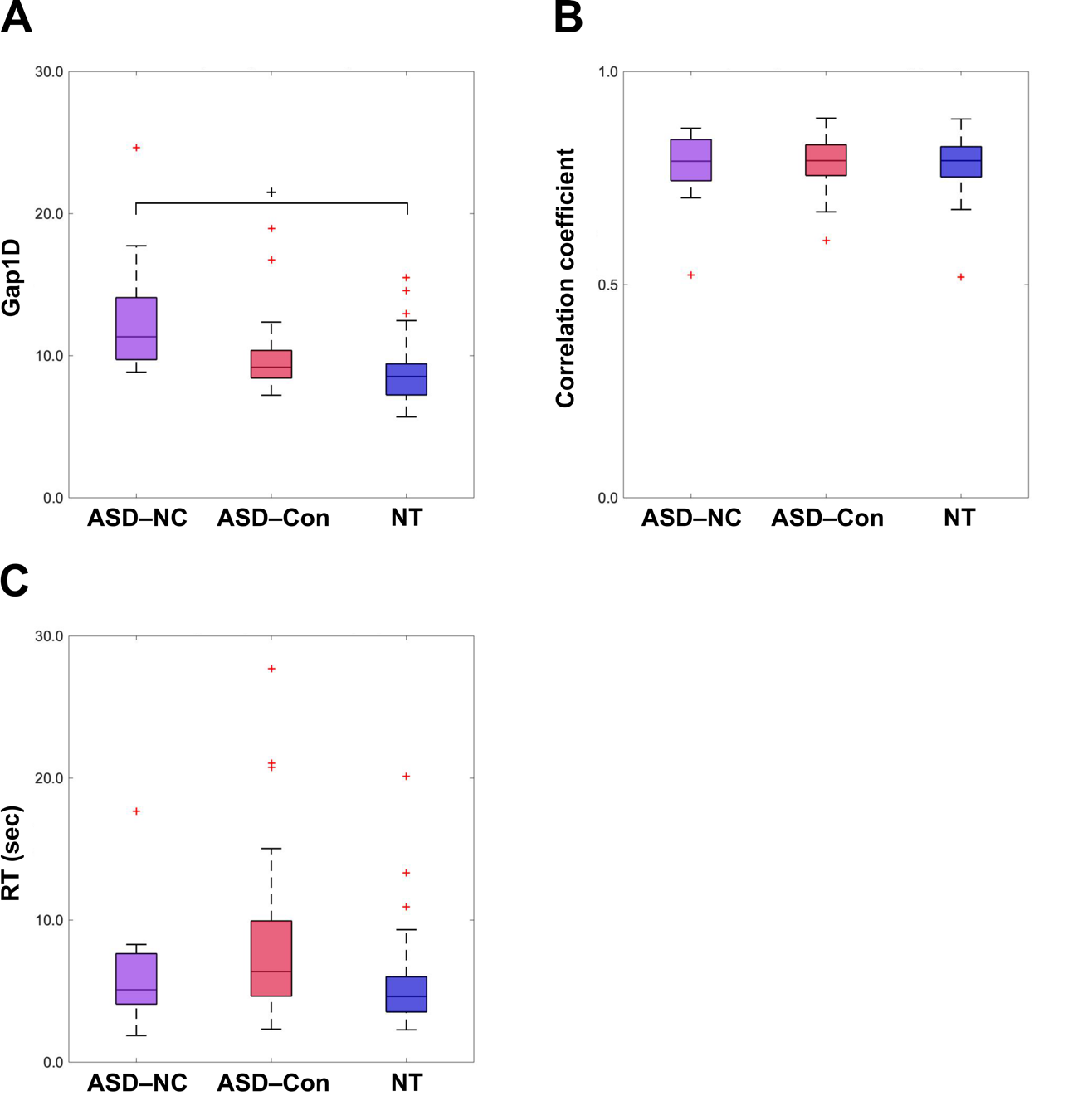


Figure S3 Exploratory analyses on the initial responses of the dot-counting task.

Comparison of initial-response performance during the dot-counting task among the ASD–never-conforming (ASD–NC), ASD–conforming (ASD–Con), and NT groups. (A) Using Welch’s one-way ANOVA, we compared the deviations of participants’ initial responses from actual number of dots (i.e., Gap1D) among the ASD–never-conforming, ASD–conforming, and NT groups. As a result, we found the significant main effect of Group (*F* (2, 18.61) = 3.55, *p* = .049, η_p_^2^ = 0.276). Games–Howell post hoc tests indicated that the ASD–never-conforming group’s initial responses deviated marginally more from the true number of dots than those of the NT group (mean difference = 4.20, *q* = 3.40, *p* = .093). No significant differences were observed for the remaining pairwise comparisons (ASD–never-conforming vs. ASD–conforming: mean difference = 3.01, *q* = 2.36, *p* = .265; ASD–conforming vs NT: mean difference = 1.18, *q* = 2.22, *p* = .272). (B) Using Welch’s one-way ANOVA, we compared the correlation coefficients between participants’ initial responses and the true number of dots across the ASD–never-conforming, ASD–conforming, and NT groups. No significant main effect of Group was observed (*F* (2, 20.23) = 0.07, *p* = .930, η_p_^2^ = 0.007). (C) Using Welch’s one-way ANOVA, we compared the response time of initial responses among the ASD–never-conforming, ASD–conforming, and NT groups. No significant main effect of Group was observed (*F* (2, 20.32) = 1.71, *p* = 0.206, η_p_^2^ = 0.144). The box indicates the interquartile range (25th–75th percentile), and the central line represents the median. Whiskers extend to 1.5 × IQR, and red crosses indicate outliers. ^+^*p* < .100.

Table S1 Results of GLMMS1 examining group differences in across-trial conformity tendencies

|  | Estimate | SE | *t* | *p* | 95% CI |
| --- | --- | --- | --- | --- | --- |
|  |  |  |  |  |  |
| Intercept | 0.00 | 0.01 | 0.00 | 1.000 | [−0.03 0.03] |
| Gap1O | 0.01 | 0.02 | 0.67 | .503 | [−0.02 0.05] |
| Gap1O:Group | −0.03 | 0.03 | -1.01 | .311 | [−0.08 0.02] |

*Note.* Gap1O: difference between first rating and others’ rating, Group: dummy code of group (ASD:1; NT:0), SE: standard error, CI: confidence interval.

Table S2 Results of GLMM1 of random slope model examining group differences in conformity tendencies between the preference rating and dot-counting tasks.

| ΔRating ~ Self1 + Gap1O + Gap1O:Task + Gap1O:Group + Gap1O:Task:Group + (1 + Gap1O \| Participant) | | | | | |
| --- | --- | --- | --- | --- | --- |
|  | Estimate | SE | *t* | *p* | 95%CI |
| Intercept | 0.00 | 0.01 | 0.00 | 1.000 | [-0.02 0.02] |
| Self1 | -0.17 | 0.01 | -20.33 | <.001 | [-0.18 -0.15] |
| Gap1O | 0.46 | 0.04 | 12.82 | <.001 | [0.39 0.53] |
| Gap1O:Task | -0.33 | 0.02 | -14.62 | <.001 | [-0.37 -0.28] |
| Gap1O:Group | -0.16 | 0.05 | -3.13 | 0.002 | [-0.26 -0.06] |
| Gap1O:Task:Group | 0.14 | 0.03 | 4.51 | <.001 | [0.08 0.20] |

*Note.* Self1: first rating, Gap1O: difference between first rating and others’ rating, Task: dummy code of task (preference rating:1; dot-counting:0), Group: dummy code of group (ASD:1; NT:0), SE: standard error, CI: confidence interval.
